# Impact of the menstrual cycle and barriers to football and futsal performance in Portuguese players: a survey-based cross-sectional study

**DOI:** 10.3389/fpsyg.2025.1576752

**Published:** 2025-03-20

**Authors:** Cristiana Santos, Mário Lopes, João Brito, Katrine Okholm Kryger, Carolina Wilke, Bruno Travassos

**Affiliations:** ^1^Department of Sport Sciences, University of Beira Interior, Covilhã, Portugal; ^2^Institute of Biomedicine, School of Health Sciences, University of Aveiro, Aveiro, Portugal; ^3^Portugal Football School, Portuguese Football Federation, Lisboa, Portugal; ^4^CIPER, Faculdade de Motricidade Humana, Universidade de Lisboa, Lisboa, Portugal; ^5^UEFA Medical, UEFA, Nyon, Switzerland; ^6^Faculty of Sport, Applied Health and Performance Science, St. Mary's University, London, United Kingdom; ^7^Research Center in Sports, Health Sciences and Human Development, CIDESD, UBI, Covilhã, Portugal

**Keywords:** women, soccer, competitive behavior, exercise, physical fitness

## Abstract

**Introduction:**

The objective of this study was to investigate the perceived impact of the different phases of the menstrual cycle on football and futsal participation and identify barriers that may limit the performance and participation of Portuguese female players across competition levels.

**Methods:**

An online survey was conducted with the following inclusion criteria: registered participation in official football or futsal Portuguese leagues, an age of 18 years or older, and perceived regular menstrual cycles. A total of 197 answers were obtained and analyzed.

**Results:**

The majority of participants were 18-25 years old (61%), and 59% played futsal. Top-tier league players represented 15% of respondents, mid-tier 26%, and low-tier 59%. For the majority of respondents, the first 3 days of menstruation were perceived as having the most negative impact (66%), with abdominal pain, bloating, and breast tenderness being the most common symptoms. Players also associated the menstrual phase with decreased performance (endurance and power) and self-confidence, whereas they generally felt more confident and motivated during ovulation. No significant differences were found between sports (football and futsal) or competition levels in the perceived impact on performance and participation (*p* > 0.05). Overall, 53% of the players reported a perceived lack of knowledge and trust in their coach and other staff members to talk openly about menstrual health. The most cited external barrier to sports participation was the fear of leaking. To address the identified challenges, five intervention levels were proposed: communication, comprehension, education, equipment, and facilities.

**Discussion:**

These findings emphasize the need for better communication, education, and structural support to reduce menstruation-related barriers.

## Introduction

Football and futsal are two team sports with a significant increase in female athlete participation worldwide. Portugal is not an exception. Recently, the Portugal Football Observatory reported that the participation of female athletes in football and futsal has increased by 132% between the 2012/2013 and 2022/2023 seasons (Portugal Football Observatory, 2024). Despite the substantial rise in participation, little is known about the menstrual health of these athletes and its impact on their participation and performance. To guarantee a sustainable future for women's football and futsal, it is crucial to enhance the understanding of the unique aspects of the women's game and the female players. This will help in continuously supporting the quality of their training and performance (de Jonge et al., 2019).

Women's menstrual health is an important factor in optimizing support for female players. Multiple studies report that the majority of women experience some type of pre-menstrual symptoms, with the most frequent being mood changes/anxiety (59–91%), increased tiredness/fatigue (80–86%), abdominal pain (71–84%), and breast pain/sensitivity (83%) (Brown et al., 2021; Bruinvels et al., 2021; Findlay et al., 2020; Morales et al., 2023). Additionally, sleep quality is often reported as being negatively affected during the pre-menstrual phase (Carmichael et al., 2021b; Ekenros et al., 2022). While no clear physical performance variations have been observed during the pre-menstrual phase (McNulty et al., 2020), several studies have highlighted changes in self-perception regarding their sporting abilities. A high percentage of female athletes report experiencing changes in training, performance, and overall wellbeing throughout the menstrual cycle (von Rosen et al., 2022). Notably, physical performance has been positively correlated with self-reported motivation and an athlete's perception of their own performance level, regardless of the menstrual phase (Dam et al., 2022). Moreover, menstrual cycle-related symptoms and wellbeing perception variations can have implications not only on individual capacity perception but also on the overall team trust climate (Read et al., 2022).

Although these topics have received the attention of the scientific community, few studies have investigated the influence of the menstrual cycle on participation and performance in female football and futsal players according to the specificities of each country and culture (Costa et al., 2022; Pinel et al., 2022). Given the cultural and contextual differences, the increasing participation in these two sports modalities (i.e., football and futsal), and despite the growing number of young female athletes in Portugal, no research has investigated the Portuguese players' perceptions and symptomatology related to the menstrual cycle. It is also essential to examine whether the differing demands of sports influence these perceptions and whether the level of competition affects those perceptions. Therefore, the primary objective of the current study was to identify the players' experienced symptoms and participation barriers related to the menstrual cycle across playing levels. The secondary objectives were to investigate differences between sports and playing levels and the influence of the most prevalent symptoms on performance perception and sports participation. We hypothesized that symptoms would be more pronounced during the menstrual phase and would impact players' experienced performance and participation. The findings from this research could provide valuable information to help break down barriers and foster an environment of trust and understanding in women's sports.

## Methodology

### Study design

A survey-based cross-sectional research was conducted according to the STROBE guidelines (Von Elm et al., 2007). This research was granted ethical approval by the Research Committee of Ethics of the University of Beira Interior (CE-UBI-Pj-2020-040). The study aligned with the Checklist for Reporting Results of Internet E-Surveys (Eysenbach, 2004).

The following inclusion criteria were set: regular participation in organized football or futsal in a Portuguese league; 18 years or older; not using oral contraceptives; and regular menstrual cycles (a menstrual cycle that occurs regularly and lasts between 21 and 35 days; Carmichael et al., 2021a). The survey was previously validated and used to analyze the impact and barriers related to the menstrual cycle in amateur female football players in England (Pinel et al., 2022). The original survey was provided by the authors in English, then translated by a football expert (BT) into Portuguese, and back-translated into English by a second author (ML) to verify congruence. The Portuguese version of the survey was piloted on two participants to assess the congruence and identify any potential linguistic errors in the questions. Since the pilot testing was informal, it may be considered a potential limitation. No changes were made following the pilot phase. Six closed-response questions concerning the influence of the technical team on the perception of sports performance impact and one open-response question regarding barriers to sports participation were added to the original questionnaire for relevance to the research, following key topics from the literature review. The questionnaire consists of 25 items related to participant characteristics, sports background, clinical history, impact of menstrual cycle on sports performance, and barriers to participation. The questionnaire was administered online via Google Forms (Google Corp, California, USA). The participation invitations were sent to the official emails of the clubs participating in Portuguese football and futsal competitions regulated by the Portuguese Football Federation, spanning low-tier (regional leagues), mid-tier (second leagues), and top-tier (the major league in the country) leagues, and no randomization was performed. Participation was voluntary, and no incentives were offered. Since the survey link was distributed to clubs, potential sampling bias was acknowledged, as it is likely that only the more engaged players answered it. Participants were provided with an information sheet at the beginning of the survey, explaining the study, and were offered the opportunity to provide informed consent before proceeding. The survey could only be accessed if informed consent was granted. The survey was divided into seven sections, with all questions being mandatory and the participants having the possibility to go back and change the answers until the survey was submitted. No identifiable information from the participants was retained. To ensure only one response per person, participants were required to complete the survey using their personal Google accounts. The response collection period lasted for 6 months (January to June 2023).

### Data analysis

The descriptive and inferential statistical analysis of the database was conducted using SPSS Statistics 27 (IBM, New York, USA). Descriptive statistic was used to describe participants' characteristics, self-reported menstrual symptoms, and experienced impact of the menstrual cycle on performance and participation. Due to sample size and observed expected counts >5, Pearson's chi-square test was used to assess the statistical relationship between the perceived impact of the menstrual cycle (“*Do you feel like your menstrual cycle limits you on playing your sport?”*) and sports type and between the level of practice and menstrual cycle characteristics. A *p*-value of ≤ 0.05 was considered for a statistically significant relationship between variables. A content analysis of open-response items was conducted using Braun and Clarke's six-step model (Braun and Clarke, 2006). The inductive analysis was performed by CS and checked again by BT, with each response grouped based on its relevance to the research. These groups were then compared and further categorized.

## Results

### Participants

A total of 197 athletes completed the survey. From these, no participants were excluded for not meeting inclusion criteria or not completing the survey. Table 1 presents the demographic and playing characteristics of the participants. The sample consisted of female football (41%, *n* = 81) and futsal (59%, *n* = 116) players playing in Portugal. The participants were distributed across different competition levels in Portugal, with 15% (*n* = 29) playing in a top-tier league, 26% (*n* = 52) in a mid-tier league, and 59% (*n* = 116) in a low-tier league. The top-tier league had the lowest representation. Participants varied in age (61%, *n* = 121 within 18–25 years, 21%, *n* = 42 within 26–30 years, and 17%, *n* = 34 within >30 years), years of football/futsal practice (10 ± 6 years), and weekly football/futsal training and match hours (51%, *n* = 100 played < 6 h, 31%, *n* = 62 played 6–8 h, 18%, *n* = 35 played >8 h).

**Table 1:** Participant characteristics.

***Characteristics***		**Football *n* (%)**	**Futsal *n* (%)**	**Total *n* (%)**
		81 (41%)	116 (59%)	**197 (100%)**
**Age (years)**	18–25	65 (54%)	56 (46%)	**121 (61%)**
	26–30	12 (29%)	30 (71%)	**42 (21%)**
	31–35	3 (16%)	16 (84%)	**19 (10%)**
	36–40	1 (9%)	10 (91%)	**11 (6%)**
	40–45	0 (0%)	4 (100%)	**4 (2%)**
**Country region**	North	23 (41%)	33 (59%)	**56 (28%)**
	Center	46 (42%)	64 (58%)	**110 (56%)**
	South	6 (30%)	14 (70%)	**20 (10%)**
	Islands	6 (55%)	5 (45%)	**11 (6%)**
**Competition level**	Top-tier	22 (76%)	7 (24%)	**29 (15%)**
	Mid-tier	27 (52%)	25 (48%)	**52 (26%)**
	Low-tier	32 (28%)	84 (72%)	**116 (59%)**
**Leagues**	Portuguese league	34 (57%)	26 (43%)	**60 (30%)**
	National league II division	16 (41%)	23 (59%)	**39 (20%)**
	National league III division	20 (95%)	1 (5%)	**21 (11%)**
	National league under 19	6 (86%)	1 (14%)	**7 (3%)**
	Regional league	5 (7%)	65 (93%)	**70 (36%)**
**Practice hours per week**	< 6	30 (30%)	70 (70%)	**100 (51%)**
	6–8	23 (37%)	39 (63%)	**62 (31%)**
	>8	28 (80%)	7 (20%)	**35 (18%)**

Total n (%) = total number of players (football n + futsal n) (%).

### Main results

#### Self-reported menstrual symptoms and signs

**Figure 1:**
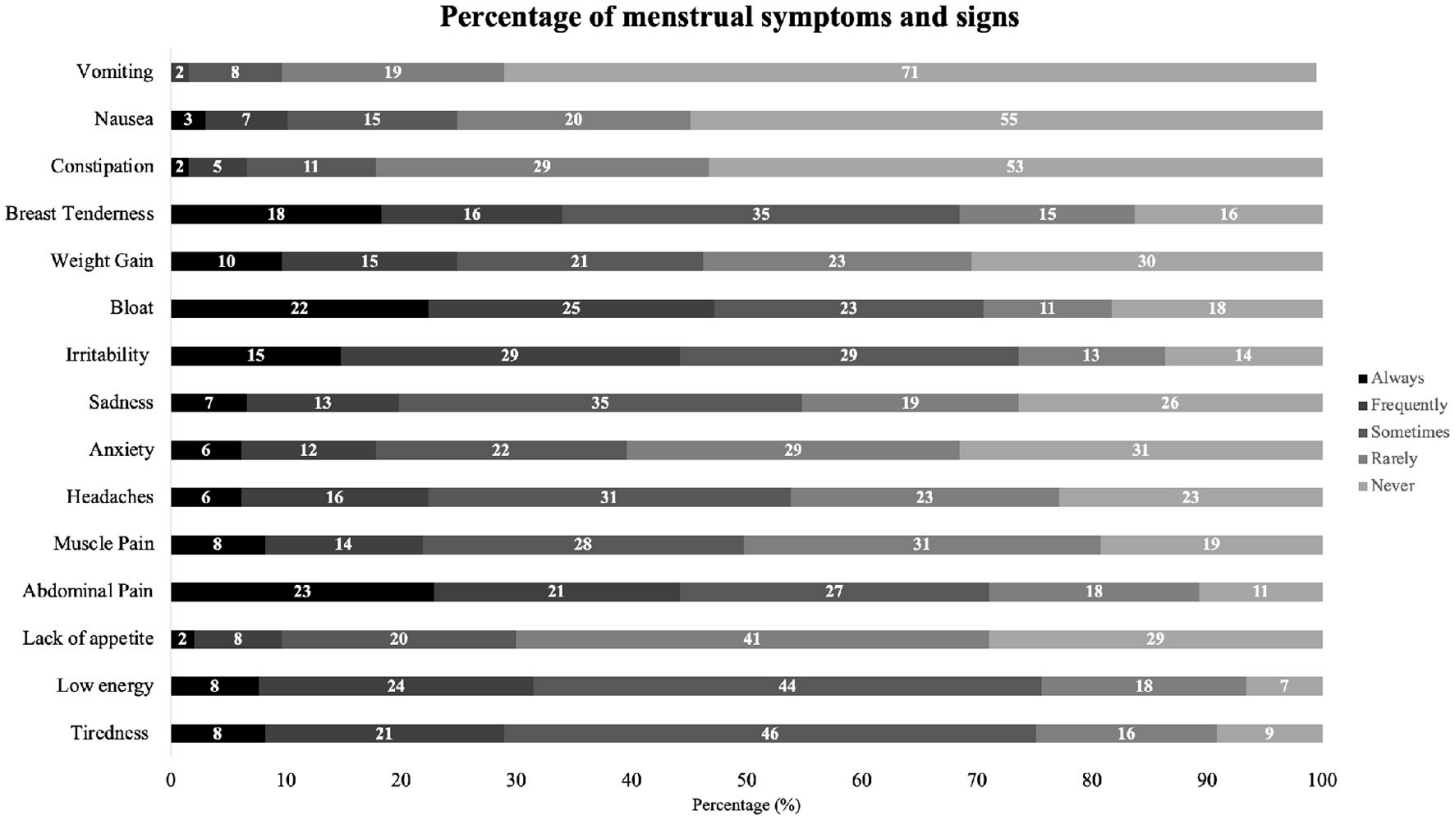
Frequency of menstrual symptoms and signs reported by participants.

When asked to report their typical menstrual flow during their menstrual phase, half of the participants reported having a moderate flow (48%, *n* = 94) and one-third reported having a heavy flow (34%, *n* = 67). Figure 1 shows the frequency of menstrual symptoms reported by the participants. The most prevalent menstruation-related symptoms were abdominal pain (23%, *n* = 45), bloating (23%, *n* = 44), and breast sensitivity (18%, *n* = 36), with fewer than one in four participants reporting these symptoms as “always” occurring. Additionally, symptoms reported as “*frequent*” included irritability (29%, *n* = 58), bloating (25%, *n* = 49), and low energy (24%, *n* = 47; Figure 1).

#### Experienced impact of menstrual cycle on performance and participation

**Figure 2:**
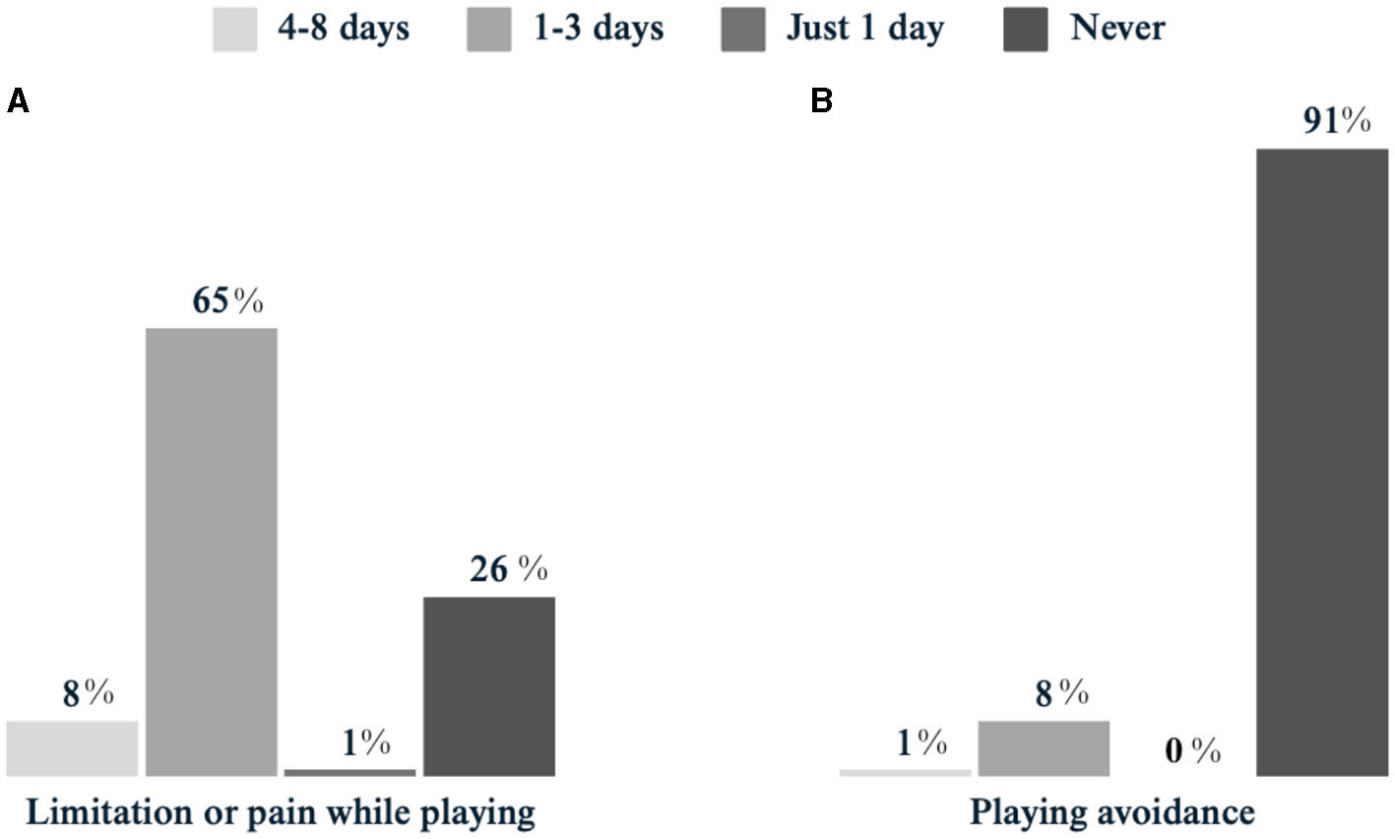
Limitation to sports participation related to menstruation. **(A)** Experienced limitation or pain while playing due to menstruation. **(B)** Playing avoidance due to menstruation.

Three in four players (75%, *n* = 147) reported feeling limited to participate in football/futsal due to menstruation. In the majority of cases, the limitation lasted for 1–3 days (66%, *n* = 128), while longer limitations were less frequently reported (8%, *n* = 15; Figure 2). Furthermore, 9% (*n* = 17) of players avoided football/futsal activities during menstruation (Figure 2). Regarding the impact on performance, less than half of the participants reported changes during the menstruation phase. Specifically, 41% (*n* = 80) experienced a decrease in overall performance, 37% (*n* = 74) reported decreased aerobic capacity, and 35% (*n* = 69) reported a decrease in power during menstruation compared to other phases of the menstrual cycle.

Although less pronounced, some participants reported lower motivation to play during the menstrual phase, with 12% (*n* = 24) indicating that it was much worse compared to the rest of the menstrual cycle. In the pre-menstrual phase, the aforementioned variables were also reported as “worse” compared to the rest of the cycle. In the ovulatory phase, one in five players reported improved confidence and power (21%, *n* = 41) compared to the rest of the menstrual cycle. Additionally, 10% (*n* = 20) of participants stated that their motivation was “*much better than in the rest of the menstrual cycle.”* Of all the variables analyzed descriptively, the one that showed the least variation across menstrual cycle phases was fear of injury, with similar percentages observed in all phases.

#### Perceived limitations to play according to sport (football and futsal) and playing levels

The analysis of the perceived impact of menstruation on participation and performance (“*Do you feel like your menstrual cycle limits you on playing your sport?”*) did not significantly differ between football and futsal players [χ^2^(4) = 6.7, *p* = 0.150] nor did it differ between playing level [χ^2^(8) = 5.9, *p* = 0.664].

#### Technical team and menstrual cycle

Among all participants, 78% (*n* = 154) were coached by a male coach. Overall, 53% (*n* = 104) felt that their coach and technical staff lacked sufficient and appropriate knowledge about the influence of menstruation on their sports performance and participation. Notably, 76% (*n* = 22) of top-tier players perceived their coaches and technical staff as having less knowledge of the menstrual cycle and its implications for sports performance compared to mid-tier (48%, *n* = 25) and low-tier players [49%, *n* = 22; χ^2^(2) = 7.279, *p* ≤ 0.05], suggesting that top-tier players were more likely to perceive a lack of menstrual cycle knowledge among their coaches and staff. One-third (32%, *n* = 62) of the players reported not feeling confident discussing the menstrual cycle with their coach and technical staff. When asked with whom they usually discuss their menstrual cycle and perceived limitations in performance and participation within their team, the majority of players (77%, *n* = 152) reported only talking to their teammates, while only 7% (*n* = 13) talked to the coach and the remaining 16% (*n* = 32) with their medical team.

#### Barriers related to sports performance and participation during menstruation

**Figure 3:**
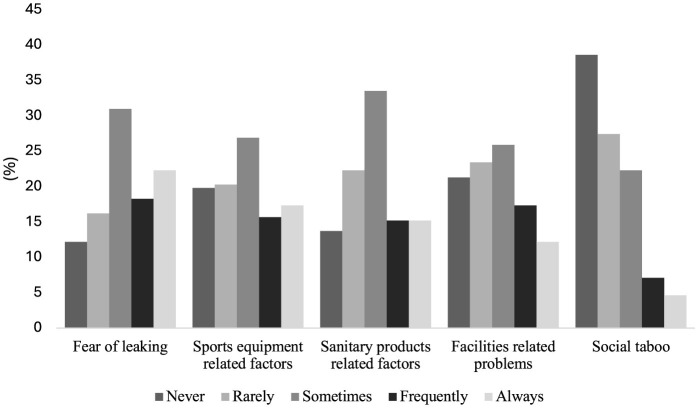
“How do you feel like the following factors affect your sports participation or performance?”

One in five responders across sports and competition levels feared menstruation leakage (“*Always*;” 22%, *n* = 44). This was followed by concerns related to the fit or color of sports equipment (”*Always*;” 17%, *n* = 34) and issues with sanitary products (“*Always*;” 15%, *n* = 30). The least influential factors affecting sports performance were the social taboo surrounding menstruation (”Never;” 39%, *n* = 76), followed by access to appropriate facilities (“Never;” 21%, *n* = 42; Figure 3).

### Proposed strategies to mitigate the barriers related to the menstrual cycle

**Figure 4:**
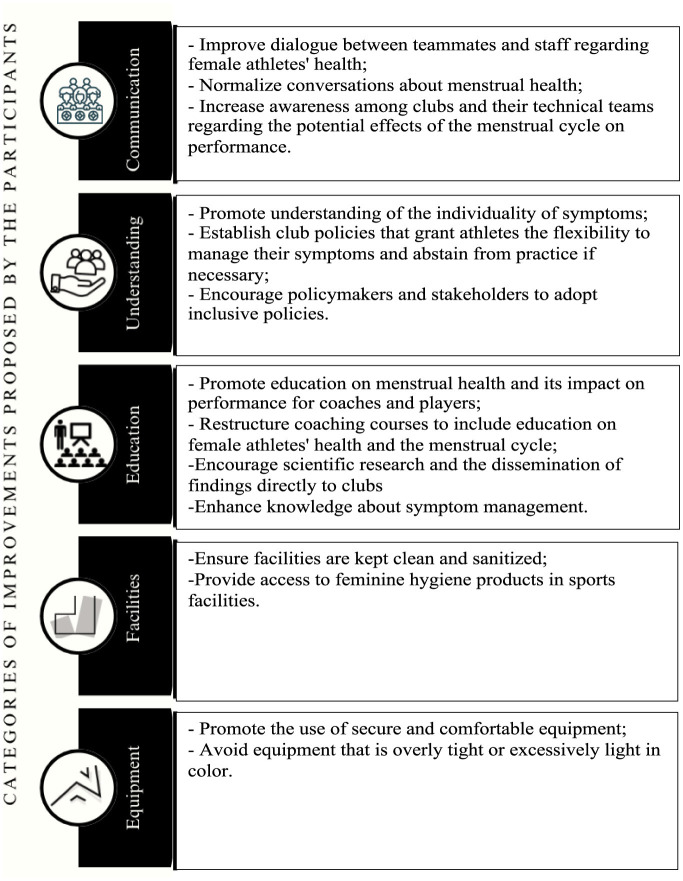
Improvements proposed by the participants to mitigate the barriers related to the menstrual cycle.

When participants were asked an open-ended question about how they thought the above-mentioned barriers could be improved, 93 responses were obtained. Examples of these responses are shown in Table 2. Five categories emerged from the responses: communication, understanding, education, equipment, and facilities (Figure 4).

**Table 2:** Strategies proposed to mitigate the barriers related to the menstrual cycle: examples of participant quotes.

**Communication**
“*We should start talking openly about the menstrual cycle with the entire team. There should be someone on the staff who discusses the importance of menstrual health, without any taboos.” “There is a need for more awareness from all parties, and the dialogue about menstrual cycle should be promoted.” “We need to improve communication between coaches and athletes, allowing the athletes to openly inform coaches about their symptoms while menstruating.” “Clubs can help normalize discussions about menstruation by making it a regular part of locker room conversations. This can help reduce the stigma surrounding menstruation and make players feel more comfortable discussing their needs.”*
**Understanding**
“*It's important to normalize menstrual tracking for athletes to ensure that adjustments to training volume and intensity can be made when needed.” “It's important for the coaches to understand the extent of the limitations each player experiences due to their individual menstrual symptoms to allow adjustments to the training plan.” “There is an important need to improve understanding, comprehension and support by encouraging open discussions and providing answers to players regarding menstrual health.” “Adopt inclusive policies: clubs should have inclusive policies that allow players to take days off during their menstrual period if necessary. This can help ensure that players have time to recover and prevent injuries.”* “*Understandig, no doubt. Especially when male coaches are leading female teams. The effects of menstruation vary from woman to woman, but men often have no idea how limiting menstruation can be for some women on certain days.”*
**Education**
“*Promotion of lectures and courses for technical teams and staffs on menstrual health.” “Facilitating access to scientific information for staff and players to improve knowledge.” “It is important for clubs to ensure that coaches and athletes have accurate knowledge about menstrual health, fostering a climate of trust and making players feel comfortable to discuss their needs.” “Provide information: t is important for clubs to offer information about menstrual health to their players. This can include details about the menstrual cycle, common symptoms, and how to manage them.” “Creating a table with each athlete's menstrual cycle information would allow the coaching staff to better understand and support players during those periods. This would help in adjusting both physical demands and psychological support according to individual needs.”*
**Equipment**
“*The selection and use of darker kits should be preferred, for primary and secondary uniforms.” “Avoid the use of white kits” “Improvements in equipment regarding the fitting and the color- shorts shouldn't be too thigh or in white colors.”*
**Facilities**
“*Ensure that the facilities are properly cleaned and that trash bins are available” “Better hygiene conditions in all sports facilities.” “Toilets should have appropriate trash bins.” “Female hygiene products should be available in the dressing rooms”*

#### Communication

Players emphasized the need to “*improve dialogue”* between teammates and technical staff regarding the menstrual cycle and its impact on sports performance. They also highlighted the importance of “*increased awareness”* within clubs and technical teams about the menstrual cycle and its potential effects on players' performance and participation. While only a few participants identified the social taboo surrounding menstruation as a performance barrier in the external barriers section, many players stressed the need to “*break this taboo”* and “*normalize the dialogue”* in their open responses. They believe this normalization would “*improve trust”* between the club, technical staff, and players.

#### Understanding

Players emphasized the importance of coaches “*understanding the individuality of symptoms”* experienced during the menstrual cycle and recognizing that availability and motivation to play may vary due to these symptoms. They also stressed that menstrual cycle information should not be used as a “*criterion for excluding players”* from selection. Additionally, players reported that both coaches and clubs should “*adopt inclusive policies”* that allow for a reduced training load when necessary and, in cases of severe symptoms, permit absences from training without repercussions.

#### Education

The most frequently mentioned topic was the importance of education. For players, education was seen as a way to develop understanding and knowledge about the impact of the menstrual cycle on sports performance and effective “*ways to manage symptoms*.” For coaches, education was suggested to improve their understanding of the individual symptoms experienced by players, allowing them to adjust training plans and manage workloads accordingly, ultimately enhancing both performance and wellbeing. Suggested methods included lectures, educational sessions, integrating this topic into coaching courses, club-level training, and creating charts to track players' menstrual cycles and associated symptoms.

#### Equipment

Players emphasized the importance of security and comfort. The results highlighted discomfort with kits that were too tight or too light in color. It was suggested that primary and secondary uniforms should not be white and that overly tight uniforms should be avoided.

#### Facilities

Hygiene conditions were identified as the main area for improvement by participants. They emphasized the need for clean and sanitized facilities, including toilets equipped with trash bins and toilet paper. Additionally, some suggested providing feminine hygiene products, such as tampons and sanitary pads, in the changing rooms.

## Discussion

This study aimed to explore the experienced symptoms and barriers to participation and sports performance related to the menstrual cycle among female football and futsal players in Portugal. The main findings indicate that (1) players perceive a negative impact on performance during menstruation, with the greatest limitations occurring in the first 3 days; (2) the most frequently reported symptoms are abdominal pain, bloating, and breast tenderness; (3) the menstrual phase is strongly associated with perceptions of decreased performance, self-confidence, endurance, power, and readiness to play, while the ovulation phase is linked to higher confidence and motivation; (4) no significant differences were found between sports and competition levels in the perceived impact on performance and participation; (5) players reported a perceived lack of knowledge and trust in their coaches and other staff members for discussing menstrual cycles openly; and (6) to address menstrual cycle-related barriers, players propose improvements in communication, understanding, education, equipment, and facilities.

Consistent with previous studies, our findings suggest that the majority of football and futsal players experience some performance variations throughout their menstrual cycle (Bruinvels et al., 2022; Carmichael et al., 2021a; Ekenros et al., 2022; Pinel et al., 2022). According to previous research by Pinel et al. (2022), some players perceived a decrease in performance in the first 3 days of menstruation. Although a range of symptoms were experienced by some players in the first 3 days, absence rates remained low. This finding also aligns with previous studies (Findlay et al., 2020; Ergin and Kartal, 2020). However, further investigation is needed to better understand the biological factors and personal reasons behind players' decisions to abstain from training or matches and to develop appropriate support strategies.

The more frequently reported symptoms related to the menstrual phase included abdominal pain, bloating, and breast tenderness. These symptoms partially align with other studies on football players (SantaBarbara et al., 2024; Pinel et al., 2022). Similarly, top-tier level football players have reported fatigue and abdominal pain as the most common symptoms, which also partially support the results of this study (Read et al., 2022). This study focused on the frequency of menstrual symptoms rather than their intensity. Future research on symptom intensity among Portuguese football and futsal players would be valuable in determining how and to what extent symptom severity impacts performance and participation. This knowledge could help develop targeted strategies to mitigate symptoms, enhance player wellbeing, and optimize performance. Additionally, further research could explore ways to mitigate these symptoms in athletes, both pharmacologically and non-pharmacologically. Deodato et al. (2023) found that physical therapy, specifically manual therapy and pelvic floor exercises, could be an effective tool for mitigating primary dysmenorrhea. Similar strategies could be investigated in team sport athletes to assess their potential benefits in improving performance perception and reducing menstrual-related discomfort.

Furthermore, consistent with previous research investigating menstrual symptoms across various sports, symptoms do not appear to differ significantly between sports and competitive levels (Ekenros et al., 2024; Brown et al., 2021; Bruinvels et al., 2021; Solli et al., 2020). It is, therefore, essential for coaches and technical staff across all levels and sports to understand the prevalence and impact of both positive and negative symptoms, enabling them to provide the necessary support for players and promote continued participation in football.

A systematic review by McNulty et al. (2020) found a trivial and non-significant change in performance during the early follicular phase, though the literature remains inconclusive. However, despite being based solely on perception, some players reported a decrease in overall performance during menstruation and an increase during the ovulatory phase. These findings have also been reported in various studies (Ihalainen et al., 2024; Pinel et al., 2022), further supporting the notion that performance variations throughout the menstrual cycle may be influenced by both physiological and psychological factors. Additionally, the positive correlation between readiness for sports participation and motivation to play may play a crucial role in shaping athletes' performance and experiences throughout the menstrual cycle (Dam et al., 2022). While the present study primarily identified a higher frequency of physical symptoms, further research should emphasize psychological symptoms and athletes' mental health to provide a more comprehensive understanding of their impact on performance and wellbeing. Hence, the coaching staff's relationship with players to comfortably communicate symptoms is essential to help adjust training, improve wellbeing strategies, and support any potential psychological barriers. This has also been reported in other studies, highlighting the need to establish structured communication pathways, considering individual needs and preferences (Taim et al., 2023).

Menstrual cycle tracking has been utilized in several studies related to menstrual health. For example, Verrier et al. (2024) found that, in elite rugby players, this method led to personal benefits by increasing the understanding of their menstrual cycle and symptoms, allowing them to respond more effectively to those symptoms. It also improved relationships, enhanced communication and interactions with coaches and support staff, and facilitated team support. Tracking the menstrual cycle created more opportunities for open conversations between athletes, coaches, support staff, and teammates. Due to its simplicity, especially through phone apps, menstrual tracking is an accessible and practical method across different playing levels. It allows athletes to record cycle length, duration, and intensity of the period, as well as the occurrence and frequency of common cycle symptoms. This helps in gaining a better understanding of the individual needs and necessities of each athlete (Roffler et al., 2024). Given its effortlessness and ease of use, even at amateur-level sports competitions, menstrual tracking could be considered an effective tool for supporting the health surveillance of female athletes.

Cultural considerations should also be taken into account, as our study focuses on female Portuguese athletes, and cultural beliefs and education regarding menstruation may influence the findings. According to Coutinho et al. (2021), 86.3% of athletes from multiple sports perceived performance variations throughout the menstrual cycle. Additionally, a high percentage of athletes (82%) reported using hormonal contraceptive methods, which aligns with the contraceptive practices commonly adopted by the Portuguese population. The menstrual patterns observed in that study are similar to those found in our research, with a significant proportion of athletes reporting menstrual pain and low energy availability during menstruation. At the time of publishing, no information was found regarding Portuguese coaches' education on menstrual health. However, based on insights gathered from athletes in the present study, we can anticipate challenges in communication and mutual discomfort when discussing menstrual cycle experiences between athletes and coaching staff.

Among external barriers to performance related to the menstrual cycle, fear of menstrual blood leaking into the equipment was considered the most limiting factor. Overall, players did not perceive their coaching staff as having sufficient knowledge or confidence to openly discuss menstrual cycle topics. Improvements in five levels were proposed: communication, understanding, education, equipment, and facilities. These are similar to external barriers related to the menstrual cycle in various studies: female players continue to report constraints in communicating with their coaches, feeling fear, discomfort, and shame when discussing the topic (Read et al., 2022). Moreover, players from this study reported perceived gaps in coaches' and technical teams' knowledge about the menstrual cycle, its symptoms, and its potential impact on performance. The issue of insufficient knowledge is widely reported, along with barriers to open communication. This may explain why players prefer discussing these issues among themselves and the low percentages of participants who talk about these issues with their coaches (Armour et al., 2020). Interestingly, this study suggests a negative association between competitive level and technical team knowledge, where at a higher competitive level, players perceive their technical teams to be less understanding of the impact of the menstrual cycle on players' performance. Future research could investigate whether players at higher levels of professionalism have a better understanding and awareness of menstrual-related issues, which may lead to the perception that the technical team's knowledge is comparatively lower. Another potential line of research could explore whether, in more competitive and professional contexts, external pressures to achieve competitive results cause technical teams to undervalue these issues. A previous study investigating the educational needs of coaches regarding the menstrual cycle highlighted the necessity of providing training and increasing their knowledge to identify and refer players with menstrual dysfunctions or other related health issues, promote collaboration among athletes, coaches, and staff, and facilitate effective communication among all parties (Clarke et al., 2021). Therefore, efforts should focus on improving existing knowledge and facilitating its dissemination among players, coaches, and other stakeholders by creating education programs centered on players and their individual needs to establish a structured support network that addresses women's health and performance (McHaffie et al., 2022).

The possibility of establishing partnerships with healthcare professionals specialized in female health should be considered to educate athletes and coaching staff about menstrual health through lectures and educational sessions. Additionally, implementing club-level policies to support and protect athletes would be highly beneficial. Furthermore, enhancing the education of futsal and football coaches by restructuring coaching courses to include topics on the menstrual health of female athletes is crucial. Currently, these topics are not part of the existing coaching curricula in Portugal. Incorporating menstrual health education would empower coaches to become active advocates for their athletes' wellbeing, fostering better support, trust, and overall health management.

### Strengths and limitations

Focusing on a single country provided valuable and actionable insights into Portuguese football and futsal. However, it is important to note that findings may differ between nations and cultures outside of Portugal. A potential underrepresentation of severe symptoms should also be considered, as the stigma surrounding menstruation may influence reporting. Furthermore, while using subjective experiences in research provides valuable perspectives, it should not be mistaken for objective perceptions, and the self-reported nature of the survey could be a source of bias. For certain measures, conducting more quantitative assessments in follow-up studies would be beneficial to gain a more comprehensive understanding of the topic. It is well acknowledged that the true identification of the hormonal phase requires physiological measures, such as blood or urine samples; however, this is considered the gold standard. We believe there is value in measuring players' experience in relation to their perceptions of their menstrual cycle based on “bleeding” and estimated date calculations. This value relates to the ecological validity of the indicators that players normally use. Furthermore, the study did not inquire about the use of comorbidities or medication used by players, which could influence the impact of their experiences.

## Conclusion

The study revealed that Portuguese female football and futsal players perceive the menstrual cycle as influencing their sports participation and performance. Specifically, participants experience a variety of symptoms, although only a minority perceive these symptoms as limiting their participation during menstruation. There was no significant association between perceived participation impact and football performance and competitive levels. The main barriers identified by participants include lack of understanding, deficits in knowledge, the need for education, and poor and ineffective communication on the menstrual cycle topic. This study could serve as a foundation for further research to gain a deeper understanding of players' symptoms, limitations, and perceptions regarding the menstrual cycle. It could provide a framework to minimize menstruation-related barriers and improve overall wellbeing. The development of policies and intervention strategies for female football and futsal players at both the club and federation levels is a step that must be taken to ensure athletes' safety, health, protection, and respect.

## Data Availability

The raw data supporting the conclusions of this article will be made available by the authors, without undue reservation.
